# WikiPathways: a multifaceted pathway database bridging metabolomics to other omics research

**DOI:** 10.1093/nar/gkx1064

**Published:** 2017-11-10

**Authors:** Denise N Slenter, Martina Kutmon, Kristina Hanspers, Anders Riutta, Jacob Windsor, Nuno Nunes, Jonathan Mélius, Elisa Cirillo, Susan L Coort, Daniela Digles, Friederike Ehrhart, Pieter Giesbertz, Marianthi Kalafati, Marvin Martens, Ryan Miller, Kozo Nishida, Linda Rieswijk, Andra Waagmeester, Lars M T Eijssen, Chris T Evelo, Alexander R Pico, Egon L Willighagen

**Affiliations:** Department of Bioinformatics - BiGCaT, NUTRIM, Maastricht University, 6229 ER Maastricht, The Netherlands; Maastricht Centre for Systems Biology (MaCSBio), Maastricht University, 6229 ER Maastricht, The Netherlands; Gladstone Institutes, San Francisco, California, CA 94158, USA; University of Vienna, Department of Pharmaceutical Chemistry, 1090 Vienna, Austria; Chair of Nutritional Physiology, Technische Universität München, 85350 Freising, Germany; Laboratory for Biochemical Simulation, RIKEN Quantitative Biology Center, Suita, Osaka 565-0874, Japan; Division of Environmental Health Sciences, School of Public Health, University of California, Berkeley, CA 94720, USA; Micelio, Antwerp, Belgium; School for Mental Health and Neuroscience, Department of Psychiatry and Neuropsychology, Maastricht University Medical Centre, 6229 ER Maastricht, The Netherlands

## Abstract

WikiPathways (wikipathways.org) captures the collective knowledge represented in biological pathways. By providing a database in a curated, machine readable way, omics data analysis and visualization is enabled. WikiPathways and other pathway databases are used to analyze experimental data by research groups in many fields. Due to the open and collaborative nature of the WikiPathways platform, our content keeps growing and is getting more accurate, making WikiPathways a reliable and rich pathway database. Previously, however, the focus was primarily on genes and proteins, leaving many metabolites with only limited annotation. Recent curation efforts focused on improving the annotation of metabolism and metabolic pathways by associating unmapped metabolites with database identifiers and providing more detailed interaction knowledge. Here, we report the outcomes of the continued growth and curation efforts, such as a doubling of the number of annotated metabolite nodes in WikiPathways. Furthermore, we introduce an OpenAPI documentation of our web services and the FAIR (Findable, Accessible, Interoperable and Reusable) annotation of resources to increase the interoperability of the knowledge encoded in these pathways and experimental omics data. New search options, monthly downloads, more links to metabolite databases, and new portals make pathway knowledge more effortlessly accessible to individual researchers and research communities.

## INTRODUCTION

**Figure 1.**
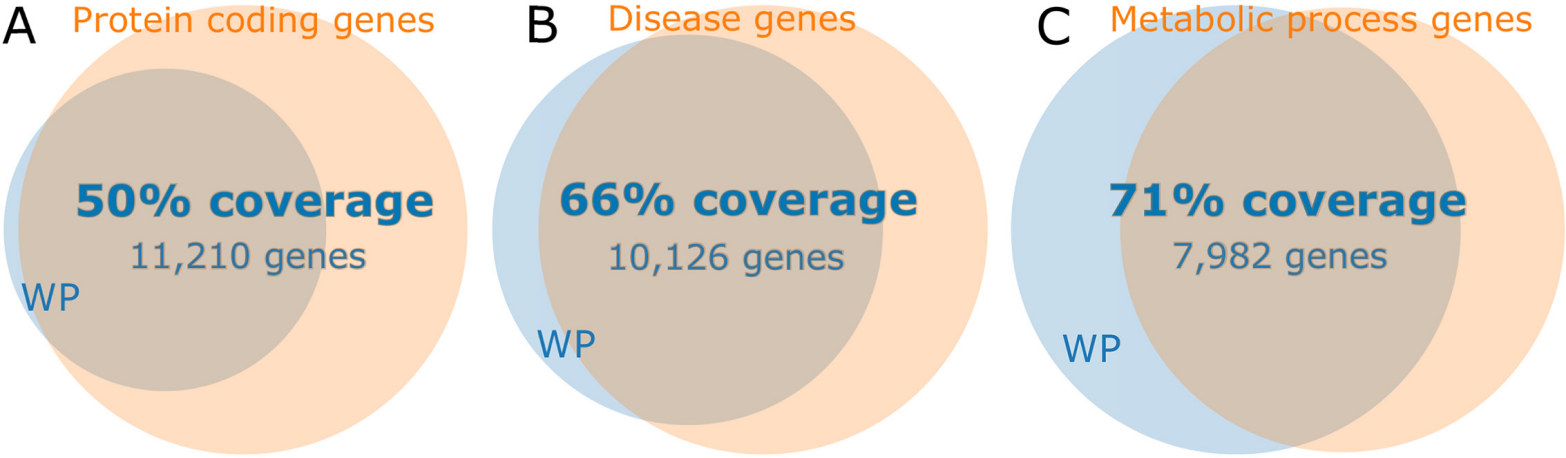
Overview of coverage of various gene spaces. WikiPathways (WP) currently covers 11 532 unique human genes. Venn diagram **A** shows that 50% of the protein coding genes (Ensembl: 22 376 genes) are found in WikiPathways. **B** shows the 66% coverage of all disease genes (OMIM: 15,262 genes), which also illustrates that the vast majority of genes in WikiPathways are associated with a disease. The **C** diagram shows that WikiPathways covers 71% of all genes known to be involved in human metabolism (GO metabolic process: 11 296 genes).

The WikiPathways initiative (wikipathways.org) started in 2008, creating a platform where pathway curation could be performed by means of crowd sourcing (1). The platform’s knowledge-base supports many life sciences communities, ranging from plant biology (2) to drug discovery (3). The use of pathway knowledge in the life sciences is wide-spread, supported by many databases (4–6) as well as integrative resources (7,8). WikiPathways has been used to analyze and integrate experimental transcriptomics, proteomics, and metabolomics data (9–12). Our previous update reported over 2300 pathways for 25 different species (13) and over the last two years this number has increased by 14% to 2614 pathways, as of September 2017. Through pathway curation and the addition of new pathways, human gene coverage present in at least one of our pathways has increased from 30% (7600) to ∼50% (11 532) of all unique human protein-coding genes, see Figure 1.

The WikiPathways database is improved by continuous data curation and updates through an expanding community: 634 individual contributors to date and 2850 edits on 1060 pathways between August 2016 to August 2017. The availability of all data under a liberal license (wikipathways.org/license) ensures that all contributors benefit from the collective effort of the full WikiPathways community. Similar to the pathway data, the code base is open source and associated formats and ontologies conform to open standards, allowing other developers to participate and join the team (github.com/wikipathways). Pathways are encoded in GPML format and created with PathVisio (14). Genes, proteins and metabolites are linked to other databases with the BridgeDb web service (15). All components are freely available, developed in open collaborations and distributed as open source or open access products.

This paper describes our efforts to increase the knowledge covered by biological pathways, with a focus on metabolites, and the usability of WikiPathways in general. These efforts include a more accurate annotation of chemical identities of metabolites, their enzymatic conversions, and protein interactions. This work was triggered by the coming of age of the metabolomics field (16), resulting in a growing amount of experimental data (17). The following sections describe our work of the past two years, organized as updates for biologists and chemists, updates for contributors and curators, and updates for bioinformaticians and data scientists.

## UPDATES FOR BIOLOGISTS AND CHEMISTS

**Figure 2.**
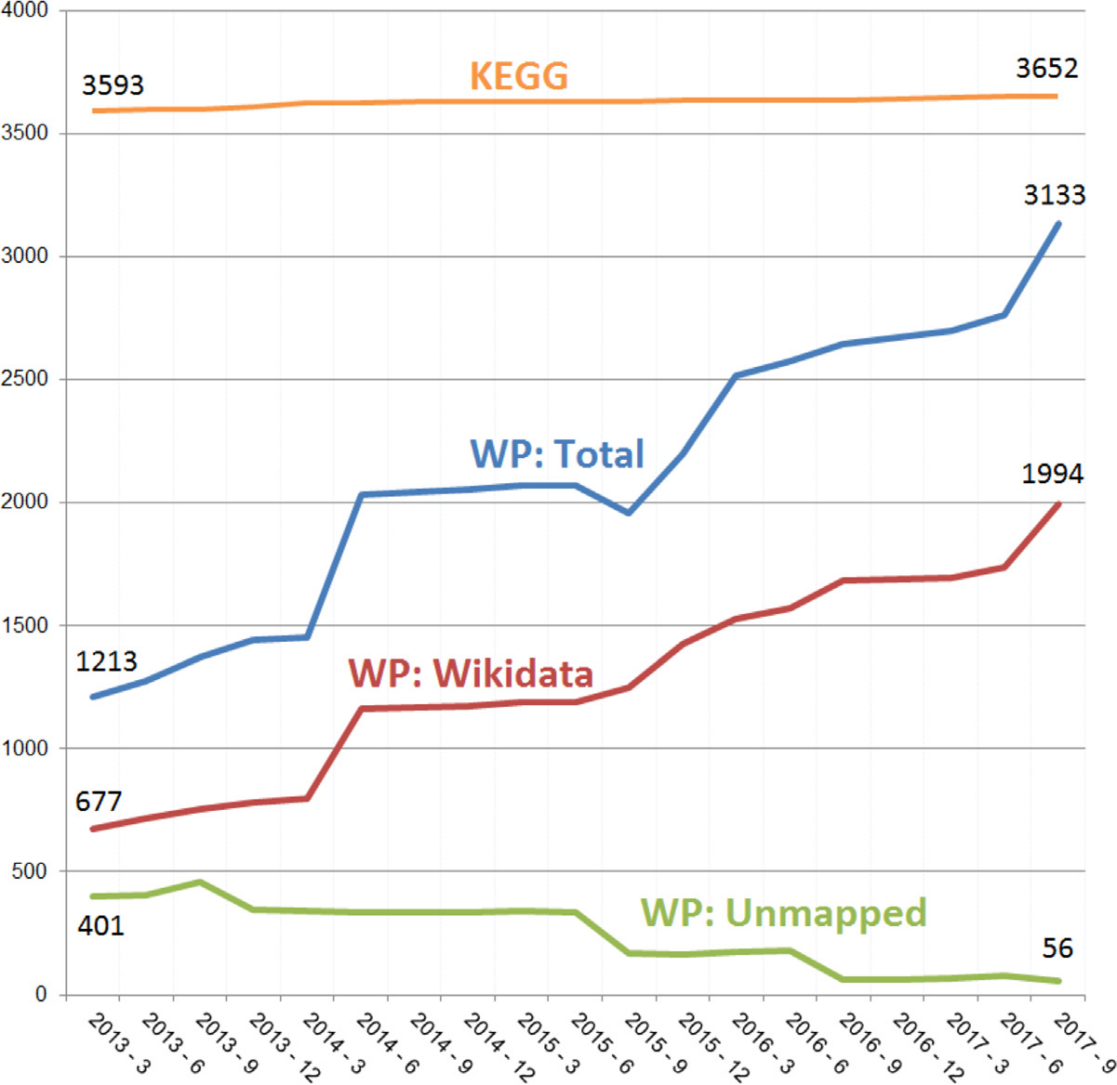
Metabolite coverage growth in WikiPathways. Taking KEGG as the standard, we plot the growth of WikiPathways (WP) coverage over the past five years. WikiPathways and KEGG unique human metabolite counts were calculated by extracting identifiers from archived WikiPathways releases and unifying to, ideally, Wikidata ID, otherwise ChEBI or HMDB. About two-third of all identifiers (blue line) could be mapped to a Wikidata (red line). Metabolite IDs that could not be mapped to these three databases, have decreased over the last five years (unmapped, green line).

To quantify the results of our curation efforts regarding the growth in metabolite data, the numbers of annotated human metabolites over the past years are shown in Figure 2. Comparing the last five years, the total number of annotated unique metabolite nodes follow a positive trend, having grown by 158% from 1213 in 2013 to 3133 in 2017. The total number of unique metabolites was estimated by counting the unique number of identifiers: all metabolite annotations were first normalized to Wikidata identifiers (18), second to ChEBI (19), third to HMDB (20), in that order, when the normalization to Wikidata was not possible. If none of these three databases provided an identifier for the listed compound, we refer to the metabolite identifier as unmapped. Importantly, the percentage of human metabolites without annotation for the full set of pathways decreased from 5% to 1% over the last five years.

Last year’s growth can partly be explained by the addition of large metabolic pathways as new content, such as the Biochemical Pathways map (Giesbertz, P., Willighagen, E. and Slenter, D. (2017) Biochemical Pathways Part I (Homo sapiens). wikipathways.org/instance/WP3604_r94542). Reactome pathways have been updated to version 61 using an extended version of the previously reported Reactome2GPML converter (21). This converter adds comments to pathway descriptions stating the original Reactome pathway ID, version, and author list.

**Figure 3.**
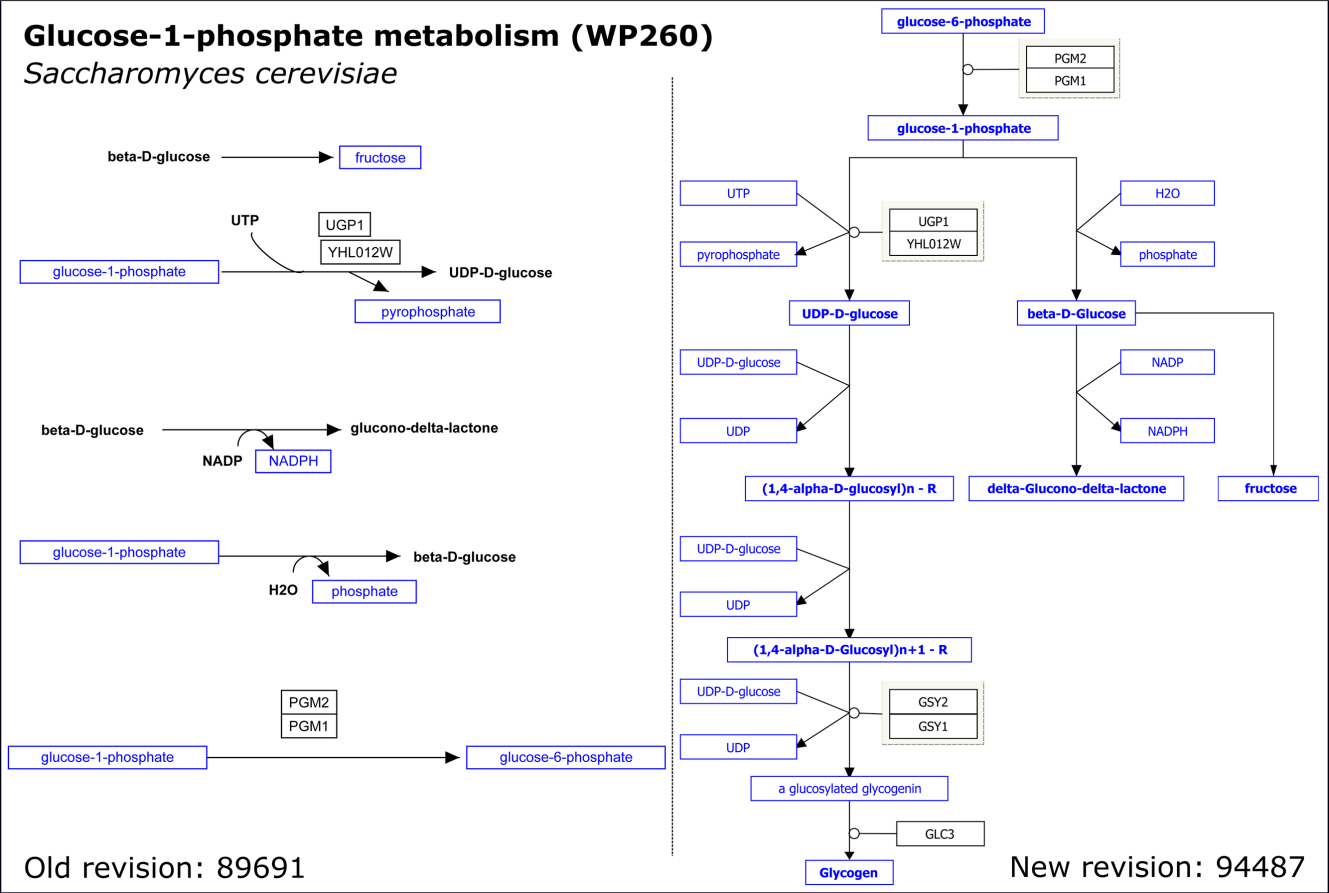
Previous and updated Glucose-1-phosphate metabolism (*Saccharomyces cerevisiae*) pathway diagram: the original pathway (left, Heckman, J., Chichester, C. and Willighagen, E. (2016) Glucose-1-phosphate metabolism (*Saccharomyces cerevisiae*). wikipathways.org/instance/WP260_r89691) represented the reactions as five separate chemical reactions and with several metabolites as text labels. After curation, the improved pathway (right, Heckman, J., Chichester, C., Willighagen, E., Slenter, D. and Kutmon, M. (2017) Glucose-1-phosphate metabolism (*S. cerevisiae*). wikipathways.org/instance/WP260_r94487) shows connected reactions with annotated metabolites.

Additionally, a considerable contribution to the growth comprises improved annotation and curation efforts. We improved the biological annotation of several small carbohydrates for multiple *Homo sapiens* pathways, such as the Glycolysis and Gluconeogenesis pathway (Username:Kdahlquist, Coort, S., Fidelman, N., van Iersel, M., Hanspers, K., Kelder, T., Bouwman, J., Pico, A., Willighagen, E., Kutmon, M., *et al.* (2017) Glycolysis and Gluconeogenesis (*H. sapiens*). wikipathways.org/instance/WP534_r94762). We now discriminate between the straight-chain (open) and cyclic (closed) forms of glucose-6-phosphate and fructose-6-phosphate and their conversions into one another. We hope that this and future work on carbohydrates in our pathways will result in a better visualization of data from the glycomics field. In order to let WikiPathways tell the full biological story of a pathway, all elements need to be specified as a gene, protein, metabolite, or pathway node, and associated with an identifier. Further curation work was aimed at improving the annotation of pathways, e.g. by annotating interactions in pathways with their directionality information, such as the direction of enzymatic reactions and the involved enzyme(s). Part of the growth, therefore, is due to converting metabolites represented as text labels to metabolite nodes. Figure 3 shows the effect of such curation on a yeast pathway.

### Links to other databases

In order to link other databases and to map experimental data onto pathways, it is crucial to annotated WikiPathways nodes with identifiers. BridgeDb is the identifier mapping tool used to translate identifiers in pathways to those in experimental data sets and third party databases (15). The pathways use various databases for annotation of metabolites, e.g. ChEBI and HMDB. In this new release, we added links to additional databases, e.g. KNApSAcK (22,23), LIPID Maps (24), and the EPA CompTox Dashboard (25). The mappings for these new databases are stored in and derived from Wikidata and ChEBI. For the EPA CompTox Dashboard alone, 36 thousand identifier mappings have been added to Wikidata to support this new link out in WikiPathways, using mapping data provided by the EPA (doi.org/10.6084/m9.figshare.3578313.v1).

### Pathway Finder tool

Besides updated content, biologists and chemists will also benefit from updates to the WikiPathways website itself. For example, a new search tool is available called Pathway Finder (wikipathways.org/pathway-finder/demo), which can be used to find pathways based on indirect queries, such as targets of specific microRNAs or which metabolites are converted by specific enzymes.

### Feedback and requests

Feedback and requests from the WikiPathways community are important to us. To facilitate more interaction between the developers, curators, authors, and users, on generic and specific questions about the WikiPathways project, tools, and pathways, we have complemented our discussion group (groups.google.com/forum/#!forum/wikipathways-discuss) with new communication channels. First, we implemented an updated request form (goo.gl/forms/FE6Ab347OLa7cBzr1), where anyone can suggest any published pathway figure to be digitized and made machine readable. We will prioritize requested pathways and assign pathway modeling tasks to people in our curation team. Given that thousands of pathway images are available, such prioritization is needed (26). Second, users can offer their general feedback in our WikiPathways User Survey (surveymonkey.com/r/wikipathways). We use the responses to gauge how WikiPathways is used in research in order to prioritize new feature development.

### Future plans

Ongoing projects extend WikiPathways to support the needs of biologists and chemists. For example, we are in the process of explicitly organizing a complete pathway archive based on ontology tags. These ontologies are sourced from the comprehensive collection of biomedical ontologies available at BioPortal (27). Future search and browse features will use ontologies to enable users to find relevant pathways. Another project works on a tool to represent pathway information regarding molecular classes and states for Cytoscape (28). First, the tool will describe the targets of microRNA in pathways, which is currently given in 109 WikiPathways, with 1067 occurrences (across all species). Second, an information class named protein modifications is added in pathways. Several pathways include protein nodes with state information, such as phosphorylation (1852 occurrences in 119 pathways) and ubiquitination (47 occurrences in 26 pathways, across all species) states. The WikiPathways app for Cytoscape will use this machine readable annotation to support data visualization on protein states in the next release, scheduled to coincide with Cytoscape 3.6.0 (28). The Pathway Finder tool will be expanded to include transcription factor targets and drug targets searches in the near future.

## UPDATES FOR PATHWAY AUTHORS AND CURATORS

### WikiPathways Academy

To support the authoring of new pathways, we created a new interactive training environment called WikiPathways Academy (academy.wikipathways.org), which aims to provide comprehensive and easy-to-use training. The Academy covers all aspects of pathway authoring, from basic concepts and terminology to various authoring tools. Designed as a step-wise path (academy.wikipathways.org/path.html), the Academy starts with a ‘Biology 101’ primer on pathway concepts, followed by a ‘Walk along a pathway’, introducing the user to reading and interpreting pathways, and then using ‘Pathway Building Blocks’ to finally build ‘My First Metabolic Pathway’. Authors are also trained on how to leverage the WikiPathways website to create an account, publish pathways and complete pathways with ontology categories, descriptions and more.

WikiPathways Academy is designed to give the user immediate feedback on training tasks, thus creating a more effective training experience. Each step in the path consists of multiple choice questions, pathway editing challenges, and WikiPathways website tasks. For editing tasks, a pathway editor is launched directly from each challenge, and results are verified automatically by a simple drag-and-drop interface. For the WikiPathways website tasks, the author performs actions at WikiPathways.org and the Academy automatically verifies each change.

### Quality Assurance protocol update

Taking advantage of the new training platform, we have redesigned our Quality Assurance (QA) protocol as an interactive experience at WikiPathways Academy, (academy.wikipathways.org/qaprotocol). The protocol consists of seven basic and five optional tasks, covering screening of new pathways and recent edits, orienting new users, fixing poorly annotated pathways and nodes, and evaluating the content of pathway collections for downstream analysis. Within each task, the curator is presented with relevant pathways one-by-one in a built-in pathway viewer, from which they can seamlessly open the pathway at WikiPathways to make necessary changes. Each task contains a detailed description of the curation steps involved, in order to assure a consistent workflow. The QA protocol is open to anyone interested in improving pathway content. We also continuously recruit a dedicated team of curators, whom rotate on a weekly basis and are displayed as the ‘Curator of the Week’ on the front page of the WikiPathways website.

### Computer-aided curation with Jenkins

The curators are further supported by automated testing. A test suite is run by a build service using the Jenkins software (jenkins.io) and uses the WikiPathways RDF that is generated from the GPML twice every day (29). The suite includes more than sixty tests using a combination of SPARQL queries and Java code to test for a variety of recurrent issues. For example, one class of tests detects the use of old data source names, such as *Kegg Compound* and *PubChem* where people should now use *KEGG Compound, PubChem-compound* or *PubChem-substance*. Other tests check identifiers for unexpected values, such as non-numerical PubMed identifiers or non-numerical identifiers for *Entrez Gene* and *PubChem-compound*. Some tests also include more specific interpretation of the data. For example, tests that ensure that genes are not annotated with metabolite identifiers, or that gene-gene interactions do not represent a gene conversion. Curators can inspect the test results at their convenience at (jenkins.bigcat.unimaas.nl), while this system also informs curators if a failure is detected via a push message.

### New portals

New portals have been set up to support specific communities. A new collaboration started with scientists from the Garvin lab at Case Western Reserve (physiology.case.edu/research/labs/garvin-lab) to develop resources for renal genomics research. As a first step, we created a new Renal Genomics portal at WikiPathways (renalgenomics.wikipathways.org), which showcases 11 pathways. This effort aims to produce high-quality pathways with tissue/cell-specificity for kidney disease and biology, and to attract researchers in this domain to collaborate on pathway modeling. As part of an ongoing collaboration, a portal was created for pathway content relevant to the National Cancer Institute’s Clinical Proteomic Tumor Analysis Consortium (CPTAC, cptac.wikipathways.org). CPTAC aims to accelerate the understanding of the molecular basis of cancer through the application of large-scale proteome and genome analysis. Pathways in the portal are annotated with post-translational modifications at the protein level (states), such as phosphorylation, methylation and ubiquitination, to facilitate data overlay and integration with other CPTAC tools.

### Future plans

First, a new tool, named Pathway Presenter (pathwaypresenter.jcbwndsr.com), has been developed to create visual presentations of the pathway diagrams from WikiPathways. With this tool, presentation slides can be created where nodes in the pathway are for example highlighted or hidden. This tool will soon be released as an integrated presentation builder and viewer launched from any pathway page at WikiPathways.org. Second, a recent analysis showed that 150 pathways have been cited in literature by their pathway identifier. We want to provide different citation styles, compatible with programs that make use of e.g. BibTeX and Endnote, for individual pathways. By adoption of data citation standards, we want to enable researchers to effortlessly and accurately acknowledge pathway authors.

## UPDATES FOR DATA MINERS AND PROGRAMMERS

### Data availability

We initiated a new archive for monthly releases of WikiPathways content at (data.wikipathways.org). Each release includes the updated contributed pathway content that has undergone community curation and internal quality assurance to ensure (i) standard identifiers for genes, proteins and metabolites, (ii) properly modeled interaction and reactions, (iii) adequate descriptions and tags, (iv) literature references and (v) ontology term annotations. These releases currently provide the pathway content in formats for computational analysis (GPML, GMT, RDF) and for visualization (SVG). Daily releases and other formats (PNG, PDF or BioPAX) are also available from the main download page (wikipathways.org/download).

Web services are available for programmatic access to WikiPathways content. We also now provide interactive Swagger documentation using OpenAPI (www.openapis.org), which is available at webservice.wikipathways.org. Matching client side libraries for Java, JavaScript, Python, and R have been updated and are available at (github.com/wikipathways/) under wikipathways-api-client-java, wikipathways-api-client-js, wikipathways-api-client-py and rwikipathways. With the use of OpenAPI tools, client code can be automatically generated for other popular programming languages, if needed.

Noteworthy is the recent license change of the WikiPathways content. After community requests to include pathway content in Wikidata, and consultation with the author community, the CC-BY license was replaced by the more liberal CCZero waiver. With this waiver, WikiPathways is placed as completely as possible in the public domain, so that others may freely build upon, enhance and reuse our works for any purposes without restriction under copyright or database law. This change enables inclusion in Wikidata and benefits other projects that reuse the pathways. The availability of the human WikiPathways in Wikidata allows enriching these pathways with the other biomedical data (30), and a few example queries available at (wikidata.org/wiki/User:Pathwaybot/query_examples) demonstrate the advantages.

### Issue tracker

In addition to the aforementioned feedback mechanisms, developers are further supported by the ability to request new features and provide feedback regarding WikiPathways via the issue tracker on GitHub (github.com/wikipathways/wikipathways.org/issues).

### Reusability of WikiPathways database

To further automate dissemination, we adopted the recently introduced FAIR principles, which propose to enhance the Findability, Accessibility, Interoperability, and Reusability of metadata (31). To achieve this goal a FAIR data point (FDP) was set up at (fdp.wikipathways.org). The FDP implements the FAIR principles by the use of rich metadata and resource description, using linked data approaches and public ontologies. The FDP describes the data resources, provides clear copyright and license information, provenance information about when the data was generated and by whom, and points to a download location. This information allows search engine crawlers to find and index the resources, implementing the findability requirement of the FAIR principles.

### Future plans

A development regarding semantic web formats is a project to create nanopublications of facts captured in WikiPathways, such as which biological entities are interacting with each other. Nanopublications have a rich provenance model, allowing stating the origin of the fact, linking to specific research papers, or even to experiments (32,33).

## CONCLUSION

The WikiPathways project is thriving. The FAIR and open science approach and the extensive community support continues to trigger growth of the project and the database content. In the following years, our growth will be supported by recently renewed funding. The updates presented here, for biologist, chemists, authors, curators, and data scientists, demonstrate the success of our approach and open up new ways in which biological complexity can be represented and reused by others. Examples of such complexity include post-translational modifications affecting protein activity and the temporal dynamics of processes. Our recent efforts around the content and curation of metabolic pathways show a high potential for adoption by the metabolism and metabolomics communities and those applying these technologies. In fact, the demonstrated continued growth in content and features since the 2016 update shows we are getting closer to reaching our commitment to capture every pathway of interest and share them in as many useful ways as possible.
